# Enucleation for Giant Liver Haemangioma

**DOI:** 10.1155/1994/83792

**Published:** 1994

**Authors:** David M. Nagorney

**Affiliations:** Mayo Clinic 200 First Street SW, Rochester MN 55905 USA


quantitative measures of liver function remained good. These data are consistent with our own study comparing DSRS and sclerotherapy[1] which measured quantitative liver function. We showed that patients successfully managed with sclerotherapy, who had no major rebleeding, showed a significant improvement in galactose elimination capacity over the first year[1]. Lesson: the data in this study show that neither of the randomized therapies significantly accelerate liver failure compared to each other.

What of the high mortality and failure to achieve surgical rescue of patients who failed sclerotherapy in this study? This factor is the major difference between this study and our previously published study[1]. In the Emory study only one patient randomized to sclerotherapy died as a direct result of rebleeding, and 12 were successfully salvaged by surgical management. In contrast, in the present study 8 patients died as a direct result of rebleeding in the sclerotherapy group and only 5 patients had surgical rescue. The authors correctly point out that access was a major problem for their sclerotherapy patients who rebleed, and this highlights one of the shortcomings of sclerotherapy. What are the lessons? In patients in whom sclerotherapy is selected as primary management, a strategy should be in place from the outset to treat rebleeding or consider surgical treatment for persistent high risk varices.

Finally, it must be remembered that sclerotherapy and surgical shunt are not the only treatments for variceal bleeding. Liver transplant has dramatically altered the management of patients with end-stage liver disease. But, it is end-stage liver disease and not variceal bleeding per se which is the indication for transplant. However, in any patient who bleeds from varices, full evaluation at the time of the initial bleed is a critical step to help answer the question: is this patient now, or will they in the future be a candidate for liver transplant? The answer to that question will influence treatment choice. We have recently published an approach to the evaluation of such patients[4]. In addition, the radiologists are back in the fray[5]. Transjugular intrahepatic portal system shunts (TIPS) can provide portal decompression, but lack of randomized trials and good objective data leave the role of TIPS to be defined.

In summary, the four prospective randomized trials which have compared DSRS to sclerotherapy all show better control of bleeding with decompressive shunt[6]. Neither therapy appears to significantly accelerate liver failure. Survival differs in these studies, and the difference depends entirely on how patients who fail sclerotherapy are managed. The important factors in this are the type of patient, their access to care, and the type of care available to the patient. To the physician making management decisions, forward planning with these factors in mind is the key.

*References*

1. Henderson, J. M., Kutner, M. H. and Millikan, W. J. *et al.* (1990) Endoscopic variceal sclerosis compared with distal splenorenal shunt to prevent recurrent variceal bleeding in cirrhosis. A prospective randomized trial. *Ann. Int. Med.*, **112**, 262–269.
2. Terblanche, J., Bornmann, P. C. and Kahn, D. *et al.* (1983) Failure of repeated injection sclerotherapy to improve long-term survival after oesophageal variceal bleeding. A five year prospective controlled clinical trial. *Lancet* **2**, 1328–1332.
3. Westaby, D. and Williams, R. (1990) Status of sclerotherapy for variceal bleeding in 1990. *Am. J. Surg.*, **160**, 32–37.
4. Henderson, J. M., Gilmore, G. T. and Hooks, M. A. *et al.* (1992) Selective shunt in the management of variceal bleeding in the era of liver transplant. *Ann. Surg.*, **216**, 248–255.
5. Conn, H. O. (1993) Transjugular intrahepatic portal-systemic shunts: The state of the art. *Hepatology* **17**, 148–158.
6. Spina, G. P., Henderson, J. M. and Rikkers, L.F. *et al.* (1992) Distal splenorenal shunt versus endoscopic sclerotherapy in the prevention of variceal rebleeding. A meta-analysis of 4 randomized clinical trials. *J. Hepatology* **16**, 338–345.

J. Michael Henderson
Department of General Surgery
The Cleveland Clinic Foundation
9500 Euclid Avenue
Cleveland, Ohio 44195

### ENUCLEATION FOR GIANT LIVER HAEMANGIOMA

### ABSTRACT

*Baer, H. U., Dennison, A. R., Mouton, W., Stain, S. C., Zimmerman, A. and Blumgart, L H. (1992) Enucleation of giant hemangiomas of the liver. Annals of Surgery, 216, 673–676.*

**Cavernous hemangiomas are the most common benign tumors of the liver. Giant cavernous hemangiomas, defined as those larger than 4 cm in diameter, can reach enormous proportions. Newer imaging modalities, although often demonstrating characteristic features that strongly suggest the diagnosis, should not be augmented by biopsy because of the risk of hemorrhage. Elective surgical resection may be indicated for symptomatic giant lesions and for those with an atypical appearance where the diagnosis is in doubt. Between October 1986 and May 1991, we treated 10 patients with giant hemangiomas by enucleation or enucleation plus resection. Median operative blood loss was 800 mL (range 200 to 3000 mL). One patient required reoperation for control of postoperative hemorrhage. Detailed pathologic examination has demonstrated an interface between hemangiomas and the normal liver tissue that allows enucleation. Enucleation is an underused procedure that if carefully performed allows resection of giant hemangiomas with a reduced blood loss and the preservation of virtually all normal hepatic parenchyma.**

KEY WORDS: Giant haemangioma, liver resection

### PAPER DISCUSSION

Hemangiomas are the most common benign noncystic tumors of the liver. Although common, they/area symp-tomatic in most patients. Only patients with symptomatic giant hemangiomas (> 4 cm) usually require resection. Baer *et al.*, have recently addressed the technical management of such patients and have clearly described enucleation as an alternative technique for successful management. Broader application of this technical approach should be considered in the surgical management of patients with symptomatic hepatic hemangiomas.

Baer *et al.*, employed enucleation in 10 patients with symptomatic giant cavernous hemangiomas. Preoperative imaging included computed tomography and selective hepatic angiography. Hemangiomas ranged from 7 to 25 cm in greatest diameter. All but two of the hemangiomas involved the central liver segments IV and V and all of the hemangiomas involved two or more liver segments. Their technique consisted of liver mobilization, extrahepatic ligation of the arterial supply to the hemangioma, and blunt dissection of the hemangioma from the normal parenchyma with suture ligation of the involved intrahepatic vessels and bile ducts. Intermittent inflow vascular occlusion was used during parenchymal dissection. Median blood loss was 800 mL (range 200 to 3000 mL). There were no deaths and only one major complication which consisted hemorrhage requiring reoperation for control. Postoperative follow-up after dismissal was not detailed.

The clinical report by Baer *et al.*, dispels the myth propagated by some surgeons that safe resection of giant hemangiomas requires complete resection with a margin of normal liver parenchyma. Fear of uncontrollable hemorrhage along the interface between the hemangioma and adjacent normal parenchyma and the concern that microscopic residual hemangioma would 'recur' or grow and cause recurrent symptoms have been used as the rationale by proponents of resection. Although Baer *et al.*, provide no data to refute the latter premise (nor was it their intention to do so), they have provided descriptive data supporting the efficacy of enucleation. They have shown pathologically that a pseudocapsule exists adjacent to the hemangioma and that it is useful clinically as a plane for excision. Although enucleation has been used selectively by others, Baer *et al.*, have shown that this technique can be used for giant hemangiomas regardless of intrahepatic location. Their technique permits preservation of all normal parenchyma. It obviates transection of adjacent parenchyma which may harbor unrecognized ducts and vessels displaced by the hemangioma which, if injured, can cause significant postoperative complications. Quite simply, the described technique permits the surgeon to safely focus on excision of the tumor with optimal preservation of adjacent liver.

Should enucleation be the technique of choice for excision of symptomatic hepatic hemangiomas? Maybe, but several factors need further analysis before general acce-ptance into clinical practice. Obviously hemorrhage is the major life threatening risk during resection of hepatic hemangiomas and Baer *et al.*, have utilized hepatic artery ligation and intermittent inflow vascular occlusion to reduce this risk. Whether either of these adjuncts to enucleation are essential remains unproven. Importantly 50% of the patients in whom enucleation was employed had an operative blood loss exceeding 1400 mL. Depending upon intraoperative hemodynamics, blood products were pro-bably transfused in some patients. Albeit infrequent, transfusion related risks can be life threatening. Ideally a surgical technique for benign disease should eliminate any transfusion risk when employed. Did the adjuncts used herein reduce blood loss? Unfortunately the data presented by Baer *et al.*, leaves this question unanswered because there are no data for comparison. Is the rationale for arterial ligation sound? Substantiation that preoperative angiography identified the exact arterial supply to the hemangioma would support their contention that extrahepatic ligation of the major hepatic arterial branch to the hemangioma is advantageous. However, that data was not presented. Moreover the concept is suspect because the classic arterial supply to hepatic hemangiomas is through multiple small peripheral arteries rather than a single dominant branch arising extrahepatically from a main lobar hepatic artery. If heman-giomas are enucleated, the arterial supply will be ligated immediately adjacent to the hemangioma anyway. Whether extrahepatic ligation confers any additional benefit is unknown. Finally Baer *et al.*, favor intermittent vascular inflow occlusion to the liver during enucleation. Unless the time for enucleation of the hemangioma from the parenchyma routinely exceeded 45–60 minutes, continuous rather than intermittent occlusion would likely be more effective in reducing blood loss because intermittent perfusion of the interface would be avoided.

Although the exact technique for resection of hemangiomas may not need to be so elaborate, the concept of broader application of local resection or enucleation is laudable. Utilization of the plane of compressed liver parenchyma adjacent to the hemangioma during continuous inflow vascular occlusion should allow enucleation to be performed safely without sacrifice of adjacent normal liver and without any permanent liver ischemia. The technique of Baer *et al.*, should be heeded. Refinements will follow.

David M. Nagorney
Mayo Clinic
200 First Street SW, Rochester
MN 55905 U.S.A

### ACUTE VARICEAL BLEEDING: SOMATOSTATIN OR SCLEROTHERAPY?

### ABSTRACT

*Shields, R., Jenkins, S. A., Baxter, J. N., Kingsnorth, A. N., Ellenbogen, S. A., Makin, C.A., Gilmore, I., Morris, A. I., Ashby, D. and West, C. R. (1992) A prospective randomised controlled trial comparing the efficacy of somatostatin with injection sclerotherapy in the control of bleeding oesophageal varices. Journal of Hepatology 16, 128–137.*

**Since previous reports have suggested that somatostatin may be of value in the control of acute variceal haemorrhage, we compared its efficacy with that of injection sclerotherapy in a randomised controlled clinical trial. Eighty consecutive patients with endoscopically-proven severe variceal bleeding were randomised to injection sclerotherapy ($n = 41$) or somatostatin ($n = 39$) given as a continuous infusion of 250 μg/h for 5 days plus daily bolus administration of 250 μg. The efficacy of injection sclerotherapy and somatostatin infusion in controlling haemorrhage and preventing rebleeding (censored at 5 days), mortality (censored at 28 days) and complications was compared. The aetiology of the portal hypertension and transfusion requirements was similar between the two groups, but there were more patients with severe liver disease (Child's C) in the somatostatin group.**

